# Passive immunization to reduce *Campylobacter jejuni* colonization and transmission in broiler chickens

**DOI:** 10.1186/1297-9716-45-27

**Published:** 2014-03-04

**Authors:** David Hermans, Katleen Van Steendam, Elin Verbrugghe, Marc Verlinden, An Martel, Tomasz Seliwiorstow, Marc Heyndrickx, Freddy Haesebrouck, Lieven De Zutter, Dieter Deforce, Frank Pasmans

**Affiliations:** 1 Department of Pathology, Bacteriology and Avian Diseases, Faculty of Veterinary Medicine, Ghent University, Salisburylaan 133, 9820 Merelbeke, Belgium; 2 Laboratory for Pharmaceutical Biotechnology, Faculty of Pharmaceutical Sciences, Ghent University, Harelbekestraat 72, 9000 Ghent, Belgium; 3 Department of Veterinary Public Health and Food Safety, Faculty of Veterinary Medicine, Ghent University, Salisburylaan 133, 9820 Merelbeke, Belgium; 4 Institute for Agricultural and Fisheries Research, Technology and Food Unit, Brusselsesteenweg 370, 9090 Melle, Belgium

## Abstract

*Campylobacter jejuni* is the most common cause of bacterium-mediated diarrheal disease in humans worldwide. Poultry products are considered the most important source of *C. jejuni* infections in humans but to date no effective strategy exists to eradicate this zoonotic pathogen from poultry production. Here, the potential use of passive immunization to reduce *Campylobacter* colonization in broiler chicks was examined. For this purpose, laying hens were immunized with either a whole-cell lysate or the hydrophobic protein fraction of *C. jejuni* and their eggs were collected. In vitro tests validated the induction of specific ImmunoglobulinY (IgY) against *C. jejuni* in the immunized hens’ egg yolks, in particular. In seeder experiments, preventive administration of hyperimmune egg yolk significantly (*P* < 0.01) reduced bacterial counts of seeder animals three days after oral inoculation with approximately 10^4^ cfu *C. jejuni*, compared with control birds. Moreover, transmission to non-seeder birds was dramatically reduced (hydrophobic protein fraction) or even completely prevented (whole-cell lysate). Purified IgY promoted bacterial binding to chicken intestinal mucus, suggesting enhanced mucosal clearance in vivo. Western blot analysis in combination with mass spectrometry after two-dimensional gel-electrophoresis revealed immunodominant antigens of *C. jejuni* that are involved in a variety of cell functions, including chemotaxis and adhesion. Some of these (AtpA, EF-Tu, GroEL and CtpA) are highly conserved proteins and could be promising targets for the development of subunit vaccines.

## Introduction

Today, campylobacteriosis is the most frequently reported zoonotic diarrheal disease worldwide, with *Campylobacter jejuni* as the major causative agent [1]. Despite many efforts aimed at minimizing *Campylobacter* contamination of poultry, no effective, reliable intervention measures exist to reduce bacterial numbers in the broiler gut [2]. In general, broiler chickens become colonized from the age of two weeks onward [3]. Transportation of immunoglobulin (Ig) Y, the major Ig class in chickens, from hen to embryo via egg yolk is believed to play a key role in the protection of young chicks with an immature immune system against *Campylobacter* colonization during two to three weeks post-hatch [4,5]. From two weeks onward, the concentration of maternally derived anti-*Campylobacter* IgY drops significantly, which coincides with an increased colonization susceptibility of the broiler chicks. Passive immunization of chicks may be prolonged by feeding broilers with high levels of anti-*Campylobacter* antibodies recovered from immunized hens [4]. Following immunization, specific IgY is induced and transferred from the serum to the egg yolk [4], where it is accumulated at high levels [6]. Indeed, pre-incubating *C. jejuni* with IgY from immunized hens has previously been shown to reduce fecal *C. jejuni* counts in broilers experimentally inoculated with this mixture [7]. In this study, also the effect of IgY on colonization in already-colonized animals was assessed. Results indicated that IgY induced only a limited therapeutic efficacy. After an initial drop, fecal *C. jejuni* numbers regained their original counts when treatment was stopped. Nevertheless, these observations indicate that IgY preparations from egg yolks could be a promising candidate to reduce *C. jejuni* colonization in broilers, but need to be optimized because no studies regarding this topic were reported since. Moreover, in the study by Tsubokura et al. [7] fecal bacterial counts were determined, which may not perfectly correlate to determining cecal counts, which is more sensitive [8]. In the past two decades, some promising observations have been reported concerning the use of chicken egg yolk Igs in animal models and humans for the prevention and treatment of other bacterial and viral diseases, including streptococci [9], *Helicobacter pylori*[10], human rotavirus, enterotoxigenic *Escherichia coli*, *Yersinia ruckeri* and *Salmonella* species [4,11]. In these studies, protection was primarily observed after infection with the homologous strain.

Passive immunization using yolk IgY thus seems a promising strategy to control *C. jejuni* colonization in broiler flocks. For this purpose, the first aim of this study was to examine whether hyperimmune egg yolk could reduce or prevent cecal *C. jejuni* colonization in broiler chicks. The second objective was to identify the *C. jejuni* antigens reacting with *C. jejuni*-specific IgY raised in chicken eggs*.*

## Materials and methods

### Experimental animals

Commercial brown Leghorn laying hens and day-of-hatch Ross broiler chickens of both sexes from a local farm were raised in group until treatment. Birds were provided with a commercial feed and water *ad libitum*. Husbandry, euthanasia methods, experimental procedures and bio-safety precautions were approved by the Ethical Committee (EC) of the Faculty of Veterinary Medicine, Ghent University, Ghent, Belgium (EC numbers: 2010/174 and 2011/029). Chicks were examined for the presence of *Campylobacter* in mixed fecal samples using standard methods as described in [12] and proved to be free of *Campylobacter*.

### Bacterial strains and culture conditions

*C. jejuni* strain KC40 from poultry origin was used for all experiments. This strain colonizes chickens to a high level [12,13]. Bacteria were routinely cultured in Nutrient Broth No.2 (NB2, CM0067; Oxoid Ltd., Basingstoke, Hampshire, UK) supplemented with Modified Preston *Campylobacter*-selective supplement (SR0204E; Oxoid) and *Campylobacter*-specific growth supplement (SR0232E; Oxoid), at 42 °C under microaerobic conditions (5% O_2_, 5% CO_2_, 5% H_2_, 85% N_2_). *C. jejuni* bacteria were enumerated by preparing 10-fold dilutions in Hank’s Balanced Salt Solution (HBSS; GIBCO-BRL, Invitrogen, Carlsbad, CA, USA) and plating on modified charcoal cefoperazone deoxycholate agar (mCCDA, CM0739; Oxoid) supplemented with CCDA selective supplement (SR0155E; Oxoid) and *Campylobacter*-specific growth supplement, followed by microaerobic incubation at 42 °C for 22 h.

### Extraction of *C. jejuni* hydrophilic and hydrophobic proteins

To prepare the immunizing agent, cells from a *C. jejuni* culture were collected by centrifugation (5000 × *g* for 30 min at 4 °C), washed in HBSS and sonicated on ice for 30 s in extraction buffer 1 (EB1; 40 m*M* Tris, supplemented with tributylphosphine solution, protease inhibitor cocktail, phosphatase inhibitors PP2 and PP3, DNase and RNase (Sigma-Aldrich, Steinheim, Germany) using a tip sonicator (Qsonica, Newtown, VS). After each sonication step, 12 in total, the sample was left on ice for 30 s. After centrifugation at 16 000 × *g* for 10 min at 4 °C the supernatant (containing the hydrophilic proteins) was collected and the pellet resuspended in extraction buffer 2 (EB2; 40 m*M* Tris, supplemented with 5 *M* urea, 2 *M* thiourea, 2% 3-[(3-cholamidopropyl)dimethylammonio]-1-propanesulfonate (CHAPS), 0.2% carrier ampholytes, 100 m*M* dithiothreitol (DTT) and the respective inhibitors as mentioned above). After sonication and centrifugation of the latter suspension at 5000 × *g* for 3 min to remove cell debris and non-lysed cells, the supernatant (containing the hydrophobic proteins) was either pooled with the first supernatant (to obtain the whole-cell protein lysate) or stored (−20 °C) separately.

### Immunization of laying hens

*Campylobacter*-free commercial Brown Leghorn chickens were randomly assigned to vaccination groups at the age of 19 weeks. Chickens were immunized with either 50 μg of the *C. jejuni* KC40 whole-cell lysate (EB1 + EB2) or 25 μg protein of the hydrophobic protein fraction only (EB2), by intramuscular injection of 250 μL of a 1:1 mixture with Freunds’ Complete Adjuvant (FCA) at four different sites of the pectoral muscle. Chicks of the control group were immunized with a mixture of HBSS and FCA. Starting from the age of 21 weeks, three booster immunizations using Freunds’ Incomplete Adjuvant (FIA) were given in a two-weekly time interval. Starting from one week after the second boost, eggs of the animals were collected and stored at 4 °C.

### Determination of egg yolk and white IgY, IgA and IgM titers

At weekly time intervals, starting from the day of the first immunization, eggs were collected from both groups. The egg yolk and white were separated and pooled per group, diluted 1:5 (wt/vol) in distilled water and mixed thoroughly. After overnight incubation at 4 °C, the supernatant, containing the water-soluble fraction of the egg yolk and white, respectively, was collected for immunoglobulin quantification using enzyme-linked immunosorbent assay (ELISA). Wells of a 96-well Nunc-MaxiSorp microtiter plate were coated with 50 μL of a suspension of *C. jejuni* KC40 cells (2 × 10^7^ cfu/mL) in coating buffer (2.16 g Na_2_CO_3_.10H_2_O + 1.935 g NaHCO_3_ in 500 mL H_2_O). Bacterial cells were either heat-killed or processed to antigenic fractions by sonication as described above. Plates were incubated overnight at 4 °C, after which the wells were washed three times with HBSS followed by a final wash step using wash buffer (WB; PBS + 0.1% Tween 20). Next, the wells were incubated with 100 μL blocking buffer (BB; WB + 1% bovine serum albumin) at room temperature (RT) for 1 h to reduce non-specific binding of antibodies. Subsequently, 100 μL of two-fold dilutions (in BB) of the supernatant were added to the wells in triplicate. The plates were incubated at RT for 90 min. After incubation, the wells were washed three times using HBSS and once using WB and 100 μL of 1/10000 (in WB) of horseradish peroxidase (HRP)-labelled anti-chicken IgY (Sigma), 1/20000 HRP-labelled goat anti-chicken IgA (Bio-Connect, The Netherlands) or 1/10000 HRP-labelled goat anti-chicken IgM (Gallus Immunotech Inc., Canada) were added to each well. After incubation and washing as described above, 50 μL of 3,3′,5,5′-tetramethylbenzidine (TMB; Sigma) substrate was added. After 10 min the reaction was blocked by adding 50 μL 0.5 *M* H_2_SO_4_ to the wells. The absorbance was then measured at 450 nm using an automated spectrophotometer. Antibody titres from egg yolks/whites of immunized hens were reported as the highest dilution where the optical density (OD) was greater than the OD + three standard deviations of wells containing yolk/white originating from birds that only received adjuvant.

### Purification of IgY fraction from egg yolks

IgY was purified from the egg yolks according to the method of Bird and Thorpe [14].

### Collection of broiler chicken intestinal mucus

Commercial and *Campylobacter*-free 14-d-old broiler chicks were euthanized and the small intestine was collected and gently rinsed with phosphate-buffered saline (PBS) to remove fecal material. The mucus was scraped from the mucosa with a glass slide covered in parafilm, diluted 1:3 with N-2-hydroxyethylpiperazine-N’-2-ethanesulfonic acid (HEPES, 25 m*M*, pH 7.4) and vortexed. The solution was centrifuged three times at 2000 × *g* for 15 min at 4 °C. The pooled supernatant containing the crude mucus was centrifuged two times more at 5000 × *g* for 15 min at 4 °C and stored at −80 °C. Protein content was determined using a Coomassie (Bradford) Protein Assay Kit according to the manufacturer’s instructions (Biorad, Nazareth, Belgium).

### Mucus adhesion test

The effect of IgY on the adherence of *C. jejuni* to intestinal mucus was examined for the homolog *C. jejuni* strain KC40 as well as for five other *C. jejuni* strains (10kf-1.16, 7P6.12, 10C-6.1, 10kf-4.12 and 10VTDD-8). Bacteria were first incubated for 30 min at 42 °C in HBSS only or HBSS supplemented with purified IgY from *C. jejuni*-immunized or HBSS/sham-immunized hens. Mucus was diluted to a final concentration of 250 μg protein/mL in coating buffer. One hundred μL were immobilized per well of a microtiter plate (Maxisorp, Nunc) and incubated overnight at 4 °C. The wells were washed three times with HBSS and saturated with 1% (wt/vol) bovine serum albumin (BSA) in HBSS for 1 h at RT. After washing the wells two times with HBSS, 100 μL of the *C. jejuni*-IgY mixture were transferred to the wells. After a 1 h incubation at 42 °C, the wells were washed 15 times to remove unbound bacteria. Wells were thereafter treated with 200 μL 0.5% (vol/vol) Triton X-100 to release adherent bacteria and incubated for 30 min at RT while shaking. Next, 300 μL HBSS were added to each well and 10-fold dilutions of the wells were titrated on mCCDA plates.

### Motility assay

To assess the influence of *C. jejuni*-specific IgY on *C. jejuni* motility of the six strains used in the study, bacteria were pre-incubated with purified IgY from yolks of either *C. jejuni*- or sham/HBSS-immunized laying hens and 20 μL were pipetted onto semi-solid Mueller-Hinton (MH) agar. After 24 h incubation at 42 °C under microaerobic conditions, the diameter of *Campylobacter* growth from both conditions was measured.

### Multilocus sequence typing

To determine the relatedness between the *C. jejuni* strains used in this study, all strains were characterized by multilocus sequence typing (MLST) according to [15]. For determination of the sequence type (ST) and clonal complex (CC) all allelic sequences were queried against the online *C. jejuni* MLST database [16].

### Effect of in-feed hyperimmune egg yolk on transmission of and cecal colonization with *C. jejuni* in two-week-old broilers

In trial 1, day-of-hatch *Campylobacter* free broiler chicks (*n* = 22) were raised in group. At 6 days of age the chicks were randomly assigned to 2 groups (*n* = 11/group) and housed in separate isolating chambers. Animals of group 1 were provided with feed containing 5% (wt/wt) egg yolk (mixed manually through the feed) from hens sham-immunized with HBSS/adjuvant, while birds of group 2 were fed 5% (wt/wt) egg yolk from hens immunized with a whole-cell *C. jejuni* lysate/adjuvant mixture. Egg yolks were added to the feed for the remainder of the experiment. Equal amounts of feed and drinking water were provided for each group during treatment and care was taken that all animals had unlimited access to the feed and water. At the age of 10 days, three chicks of both groups were orally inoculated with approximately 8 × 10^3^ cfu of *C. jejuni* strain KC40. At 13 days of age all animals were euthanized (as described above) and the ceca as well as their contents were collected for *C. jejuni* enumeration (see below).

In trial 2, day-of-hatch broiler chicks (*n* = 54) were raised in group. At 6 days of age the chicks were randomly assigned to 6 groups (*n* = 9/group) and housed in separate isolating chambers. Animals of groups 1, 2 and 3 were provided with feed containing 5% (wt/wt) egg yolk from hens immunized with HBSS/adjuvant, while birds of groups 4, 5 and 6 were fed 5% (wt/wt) egg yolk from hens immunized with the hydrophobic protein fraction of *C. jejuni* mixed with adjuvant. At the age of 10 days, three chicks of each group were orally inoculated with approximately 3 × 10^4^ cfu of *C. jejuni* strain KC40. At 13 days of age all animals were euthanized (as described above) and the ceca as well as their contents were collected for *C. jejuni* enumeration (see below).

### Cecal *Campylobacter jejuni* enumeration

Ceca and contents were cut into small fragments, weighed, and diluted 1:9 (wt/vol) in NB2 with supplements. After homogenization, a 10-fold dilution series was made in HBSS. Of each dilution, 100 μL were spread on mCCDA plates. After 22 h incubation at 42 °C under microaerobic conditions, characteristic colonies were counted. For enrichment, diluted cecal samples in NB2 were incubated at 37 °C under microaerobic conditions. After 24 h, samples were plated on mCCDA and incubated at 42 °C in a microaerobic environment. After 24 and 48 h plates were examined for the presence or absence of *C. jejuni*.

### Two-dimensional gel-electrophoresis and Western blot analysis

To identify the immunodominant antigens of *C. jejuni* recognized by yolk IgY of immunized hens, the whole *C. jejuni* lysate was separated by iso-electric focussing (pH 3–10), followed by sodium dodecyl sulphate-polyacrylamide gel electrophoresis (SDS-PAGE) with a 4% stacking gel and 10% separating gel at 150 V for 30 min and 200 V for 60 min as previously described [17]. The separated proteins were transferred electrophoretically (50 V) onto nitrocellulose membranes (Bio-Rad) for 3 h. After washing with milliQ water, the membrane blots were blocked in 5% (wt/vol) milk in PBS for 1 h at RT. Subsequently, the blots were incubated overnight with purified egg yolk IgY, diluted to 1:5000 (vol/vol) in 5% (wt/vol) milk. After washing with 0.05% (vol/vol) Tween-20, the blots were incubated with horseradish peroxidase-conjugated goat-anti-chicken immunoglobulin G at a dilution of 1:50 000 in 5% (wt/vol) milk for 1 h. The blots were washed and proteins were immunodetected by enhanced chemiluminescence, using Supersignal West Dura Extended Duration Substrate (Pierce), and scanned and digitized using the Versa Doc imaging system. Blotting experiments were performed in duplicate to identify antigens recognized by IgY in yolk from immunized/sham-immunized laying hens.

### In-gel digestion and mass spectrometric analysis

Spots of interest (*C. jejuni* antigens reacting with hyperimmune egg yolk IgY) were cut out of the gel and processed as previously described [18]. Prior to mass spectrometry the isolated peptides were separated on a U3000 nano-HPLC device (Dionex) as previously described [19].

Identification of the peptides was performed on an electrospray ionization quadrupole time of flight (ESI-QUAD-TOF) spectrometer (Ultima, Waters). Peptides were acquired in the range of 450 to 1650 m/z in MS. The seven most abundant multiply charged ions with a minimum intensity of 60 counts per second were subjected to MS/MS (between 50 and 2300 m/z). m/z ratios selected for MS/MS were excluded for 150 s.

Data analysis was performed against the *Campylobacter* protein databank (124864 sequences) from NCBI using the in-house search engine Mascot Daemon (2.3, Matrix Science, London, UK). Carbamidomethyl (N-term), deamidation (NQ) and oxidation (M) were set as variable modifications. Peptide mass tolerance and fragment mass tolerance were set at 0.35 Da and 0.6 Da, respectively. Maximum two miss cleavages were allowed. Proteins were only considered to be positively matched if the significance was below 0.01 (*p* ≤ 0.01) and at least one peptide passing the required bold red criteria from Mascot Daemon, indicating that at least one peptide had rank 1 and a significance below 0.01.

### Statistical analysis

Data were analyzed by SPSS 17.0 software for Windows. The significance level **α** was set at 0.05. *Campylobacter* counts were first transformed to log_10_ counts before statistical analysis. For the in vivo trials, a non-parametric Mann–Whitney *U* test was carried out to compare the means of log_10_ transformed counts in chicken cecal contents (of seeders, contact animals or both) of all groups (treated and control groups). For the mucus adhesion test, a one-way analysis of variance (ANOVA) was carried out to compare the means of log_10_ transformed adherent bacteria of all groups (treated groups and control groups). Significant differences were assessed by Bonferroni Post Hoc tests. *P*-values below 0.05 were considered significantly different.

## Results

### Determination of antibody titers from egg

*C. jejuni*-specific IgY were detectable in egg yolk of hens immunized with a *C. jejuni* lysate, resulting in titers of up to 1:16000 as determined by ELISA (results not shown). In contrast, *C. jejuni*-specific IgY in egg white and *C. jejuni*-specific IgA and IgM in egg white and yolk were not detectable after immunization.

### In vitro anti-*Campylobacter* properties of egg yolk IgY

**Figure 1:**
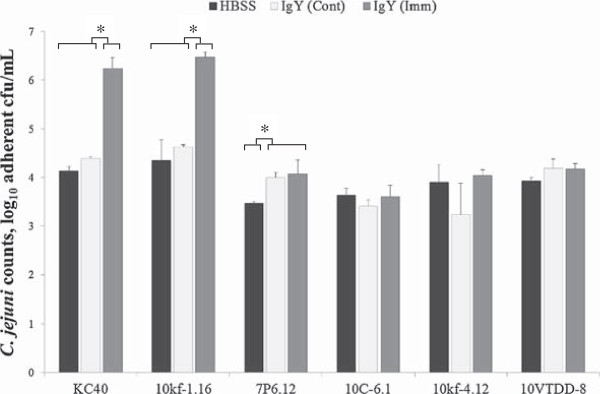
***Campylobacter jejuni *binding to chicken intestinal mucus.** Total adherent *Campylobacter jejuni* bacteria to chicken intestinal mucus are shown after pre-incubation with HBSS (control, black rectangle symbol) or IgY derived from HBSS/sham-immunized (white rectangle symbol) or *C. jejuni*-immunized hens (grey rectangle symbol). Values are represented as log_10_ cfu adherent bacteria/well. Statistical differences are denoted with an asterisk (*p* < 0.05).

Pre-treatment with purified IgY from yolks of *C. jejuni* KC40-immunized hens significantly (*p* < 0.05) promoted bacterial binding of the homologous *C. jejuni* strain to chicken intestinal mucus by approximately log_10_ 2 compared to IgY from HBSS/sham-immunized (control) animals (Figure 1). Increased bacterial binding to mucus after pre-treatment with KC40-derived IgY compared to control IgY was also observed for *C. jejuni* strain 10kf-1.16 (*p* < 0.05), whereas the binding capacity of the other four *C. jejuni* strains (7P6.12, 10C-6.1, 10kf-4.12 and 10VTDD-8) remained unaltered (*p* > 0.05). All six *C. jejuni* strains tested belong to a different clonal complex, as proved by MLST analysis (Table 1), indicating that *C. jejuni* strain KC40 and *C. jejuni* strain 10kf-1.16 are “true” heterologs. *C. jejuni* KC40-specific IgY did not affect *C. jejuni* motility of any of the tested strains (data not shown).

**Table 1:** **Multilocus sequence type and clonal complex attribution of *C. jejuni *** **strains used in the study.**

			**MLST allelic profile**						
**Isolate**	**Sequence type**	**Clonal complex**	***aspA***	***glnA***	***gltA***	***glyA***	***pgm***	***tkt***	***uncA***
KC40	794	677	10	81	50	87	120	76	52
10kf-1.16	267	283	4	7	40	4	42	51	1
7P6.12	464	464	24	2	2	2	10	3	1
10C-6.1	305	574	9	53	2	10	11	3	3
10kf-4.12	51	443	7	17	2	15	23	3	12
10VTDD-8	905	NA^1^	2	15	4	3	154	25	35

^1^not assigned.

### In vivo anti-*Campylobacter* properties of egg yolk IgY

**Figure 2:**
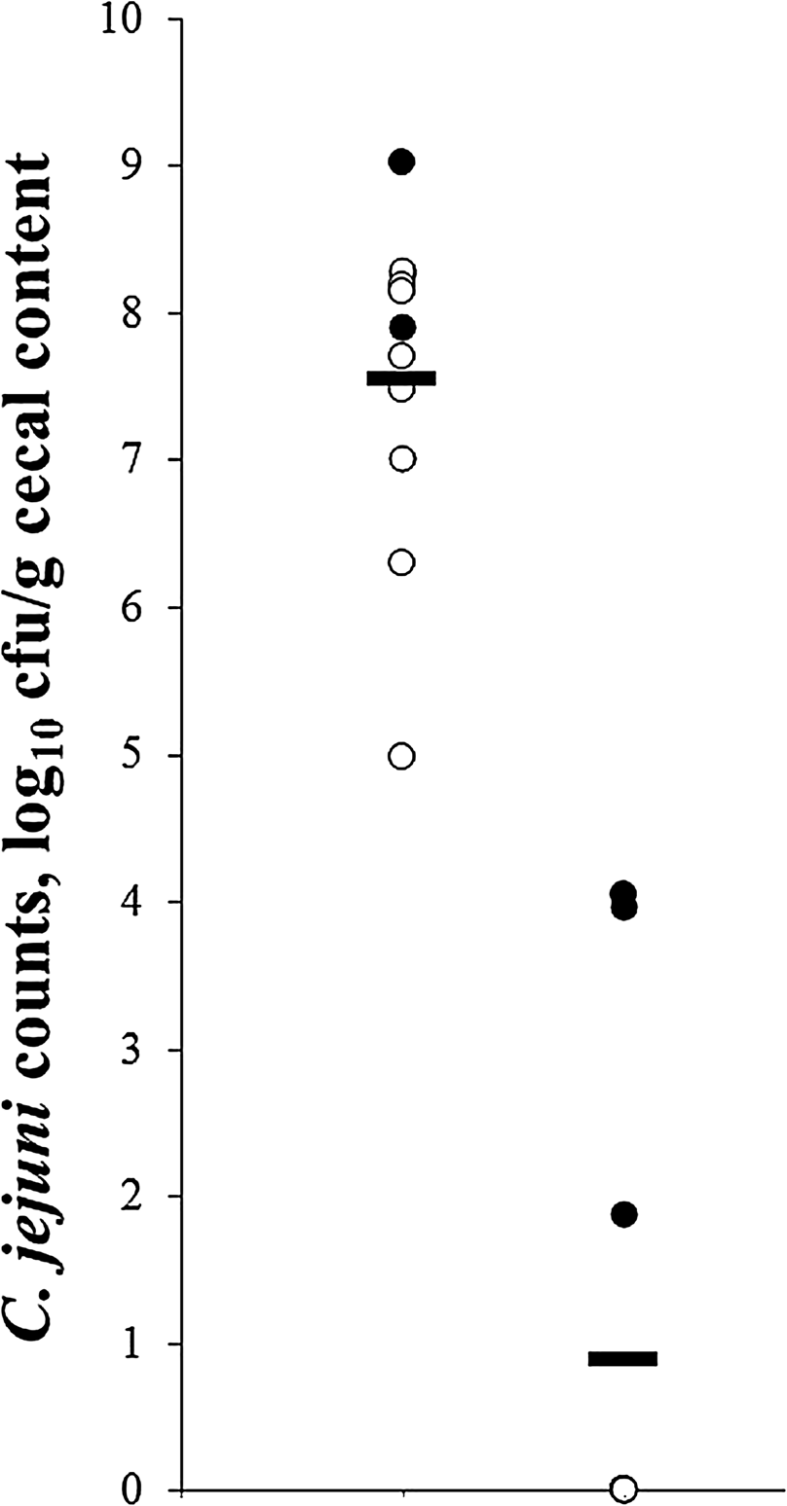
**Reduced cecal *Campylobacter jejuni *colonization with yolks from hens immunized with a whole-cell lysate of *C. jejuni*.** Individual and mean (▬) cecal *C. jejuni* counts of two-week-old co-housed broiler seeders (●) and sentinels (○) receiving standard feed supplemented with 5% (wt/wt) egg yolk from either HBSS/sham-immunized (left) or *C. jejuni* immunized (right) layers, from day six until the end of the experiment, are shown. Values are represented as log_10_ cfu/g cecal content. At 10 days of age, seeder animals were inoculated with approximately 8 × 10^3^ cfu of *C. jejuni* KC40.

In in vivo trial 1, the overall cecal *C. jejuni* numbers (Figure 2) of inoculated (seeder) broilers receiving hyper-immune egg yolks from *C. jejuni* whole-cell lysate-immunized layers were reduced by > 5 log_10_ cfu (*p* < 0.01) compared to control seeders, receiving yolks from HBSS/sham-immunized hens (3.3 ± 1.2 vs. 8.4 ± 0.6 log_10_ cfu/g cecal contents). Moreover, transmission to non-seeder (contact) chicks was completely prevented, in contrast to the control group where seeder and non-seeder birds (8.4 ± 0.6 vs. 7.3 ± 1.1 log_10_ cfu/g cecal contents) were colonized to a similar (*p* > 0.05) degree. Together, this lead to a dramatic reduction (*p* < 0.001) in the overall cecal *C. jejuni* count by log_10_ 6.7 for birds receiving hyperimmune yolk (0.9 ± 1.6 log_10_ cfu/g cecal contents) compared to birds receiving control yolks (7.6 ± 1.1 log_10_ cfu/g cecal contents).

**Figure 3:**
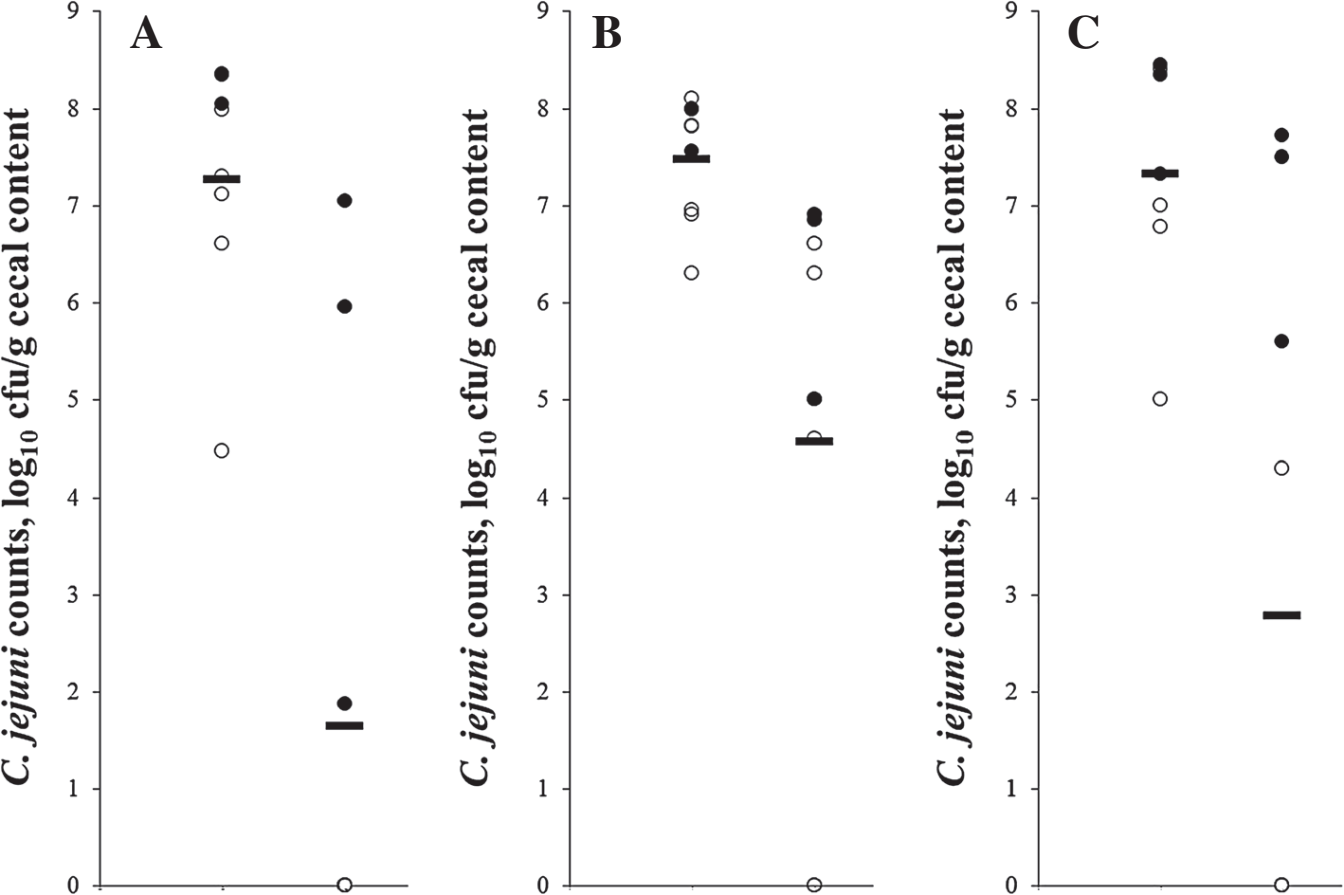
**Reduced cecal *Campylobacter jejuni *colonization with yolks from hens immunized with *C. jejuni *hydrophobic proteins.** Individual and mean (▬) cecal *C. jejuni* counts of two-week-old co-housed broiler seeders (●) and sentinels (○) receiving standard feed supplemented with 5% (wt/wt) egg yolk from either HBSS/sham-immunized (left) or *C. jejuni* immunized (right) layers, from day six until the end of the experiment, are shown. Values are represented as log_10_ cfu/g cecal content. At 10 days of age, seeder animals were inoculated with approximately 3 × 10^4^ cfu of *C. jejuni* KC40. The experiment was performed in triplicate and results of all replicates are shown **(A-C)**.

In in vivo trial 2, the protective effect of IgY induced against the outer-membrane proteins of *C. jejuni* was investigated. In two of the three replicate experiments (Figure 3A and B) a significant (*p* ≤ 0.05) reduction in cecal *C. jejuni* counts was observed in seeder animals (by respectively 3.3 and 1.6 log_10_ cfu/g cecal contents) fed hyperimmune egg yolk compared to control birds. In the third replicate (Figure 3C), counts were reduced (*p* > 0.05) by 1.1 log_10_ cfu. In replicate 1, transmission to contact birds was completely prevented, while in replicate 2 and 3 cecal counts were significantly reduced by respectively 3.6 and 6.1 (*p* < 0.01) log_10_ cfu/g cecal contents) when birds were fed hyperimmune egg yolk. Together, this resulted in an overall reduced cecal *C. jejuni* count in the replicate experiments of respectively 5.6 (*p* = 0.001), 2.9 (*p* = 0.001) and 4.5 (*p* < 0.05) log_10_ cfu/g. Averaged over three replicates, cecal *C. jejuni* numbers of inoculated broilers receiving hyper-immune egg yolks from layers immunized with the hydrophobic protein fraction of *C. jejuni* were reduced by two log_10_ cfu (*p* = 0.002) compared to control animals (6.0 ± 1.8 vs. 8.0 ± 0.4 log_10_ cfu/g cecal contents), while transmission to contact birds was greatly (*p* < 0.001) reduced (1.5 ± 2.5 vs. 7.0 ± 1.1 log_10_ cfu/g cecal contents). Together, this lead to a dramatic reduction (*p* < 0.001) in the overall cecal *C. jejuni* count by log_10_ 4.4 for broilers receiving hyperimmune yolk (3.0 ± 3.2 log_10_ cfu/g cecal contents) compared to birds receiving control yolks (7.4 ± 1.0 log_10_ cfu/g cecal contents).

### Identification of immunodominant *C. jejuni* KC40 antigens

**Figure 4:**
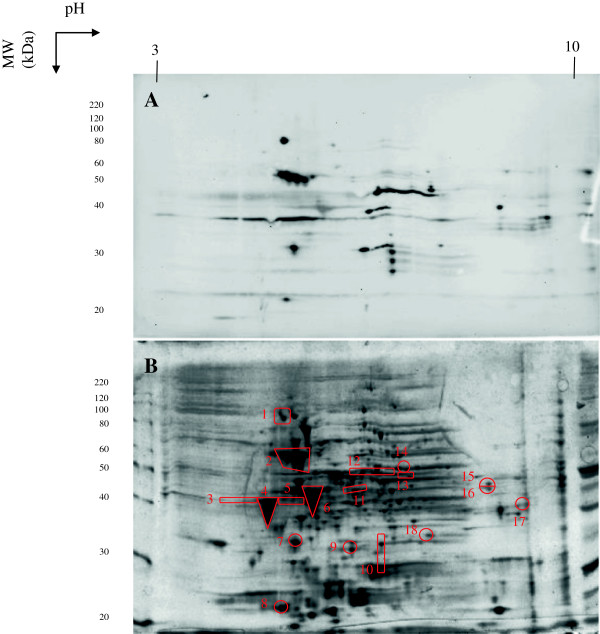
**Western blot reacted with yolk immunoglobulin Y of hens immunized with a whole-cell *C. jejuni *lysate (A) and *Campylobacter jejuni *KC40 2D-proteome profile (B).** A total protein extract of *C. jejuni* KC40 was separated by 2-dimensional gelelectrophoresis and separated proteins were detected by SYPRO® RUBY Protein staining. Immunoreactive antigens are indicated with a number and were excised from the gel and sequenced using mass spectrometry. Identified proteins are indicated by the spot numbers given in Table 2.

Western blots from 2-dimensional separated *C. jejuni* proteins immunostained with IgY from egg yolk of *C. jejuni* whole-cell lysate-immunized laying hens revealed several immunodominant antigens (Figure 4A), while blots stained with IgY from HBSS/sham-immunized hens showed only marginal reactivity (data not shown). Spots that were preferentially present on the Western blot immunostained with purified IgY from yolks of laying hens immunized with *C. jejuni* were linked to the 2-dimensionally separated *C. jejuni* proteins on the gel (Figure 4B), digested in peptides with trypsin and subjected to mass spectrometric (MS) analysis. Proteins with a Mascot score of < 56 (*p* < 0.01) or present in only one of duplicate experiments were excluded from the study. MS analysis identified several of the immunodominant antigens of *C. jejuni* KC40, represented in Table 2.

**Table 2:** **Predicted immunodominant *Campylobacter jejuni *** **KC40 antigens identified with HPLC-MS/MS.**

**Spot no.**	**Protein name**	**Gene**	**Mascot score**	**MW (kDa)**	**pI**
1	Flagellar hook protein	*flgE*	1928	90.1	4.85
2	Flagellin subunit protein A	*flaA*	4958	59.2	5.7
	Co-chaperonin GroEL	*groEL*	3281	58	5.02
	Flagellin subunit protein B	*flaB*	1476	59.4	5.66
	Flagellin subunit protein B	*flaB*	1048	26.3	5.52
3	Flagellin subunit protein A	*flaA*	693	58.8	5.53
	Co-chaperonin GroEL	*groEL*	493	58	5.02
4	Major outer membrane protein	*omp*	3071	43.1	4.54
	Glutamate-1-semialdehyde-2,1-aminomutase	*hemL*	356	46.5	8.92
	Glutathionylspermidine synthase family protein		164	44.8	4.59
5	Major outer membrane protein	*omp*	1011	43.1	4.54
6	Translation elongation factor thermo unstable	*eF-Tu*	2172	43.6	5.11
	ATP-dependent Clp protease, ATP-binding subunit	*clpX*	311	46.1	5.21
	Major outer membrane protein	*omp*	66	36	4.53
7	Chemotaxis protein V	*cheV*	1552	35.8	4.92
	Translation elongation factor thermo unstable	*eF-Tu*	461	45	5.43
	Glycyl-tRNA synthetase alpha subunit	*glyQ*	148	24.3	4.6
	Phosphoribosylformylglycinamidine cyclo-ligase	*purM*	70	36	5.07
8	FKBP-type peptidyl-prolyl cis-trans isomerase	*slyD*	743	20.5	4.75
	Chemotaxis protein V	*cheV*	330	35.8	4.92
	Inorganic pyrophosphatase	*ppa*	308	19.4	4.79
	Translation elongation factor thermo unstable	*eF-Tu*	226	43.6	5.11
	Heat shock protein GrpE	*grpE*	168	20.2	4.79
9	Translation elongation factor thermo unstable	*eF-Tu*	96	43.6	5.11
10	Branched-chain amino acid aminotransferase	*ilvE*	551	33.1	6.12
	Enoyl-(acyl-carrier-protein) reductase	*fabI*	354	30	5.67
	Putative methyltransferase		174	29.8	5.92
11	Methyl-accepting chemotaxis protein		811	40.6	5.5
	Succinyl-CoA synthetase beta chain		502	41.9	5.61
	Cysteine desulfurase, putative		472	43.2	5.83
	Phosphoglycerate kinase	*pgk*	216	43.9	6.07
12	Conserved hypothetical protein		568	53	6.28
	Pyruvate kinase	*pyk*	420	54	5.68
	Glutamyl-tRNA synthetase	*gltX-1*	306	53.3	6.19
	ATP synthase F1, alpha subunit	*atpA*	343	53.4	5.73
	Trigger factor	*tig*	255	50.9	5.69
	Aspartate ammonia-lyase	*aspA*	219	52.1	5.5
	Fumarate hydratase	*fumC*	131	23.2	6.05
13	ATP synthase F1, alpha subunit	*atpA*	856	53.4	5.73
	GMP synthase (glutamine-hydrolyzing)	*guaA*	580	57.4	6.19
14	ATP synthase F1, alpha subunit	*atpA*	1014	53.4	5.73
	Peptide transport system substrate-binding protein		488	58.7	6.52
	Acetyl-CoA carboxylase, biotin carboxylase	*accC-2*	128	54.9	6.4
15	Putative secreted carboxyl-terminal protease	*ctpA*	1607	48.3	8.7
16	Putative secreted carboxyl-terminal protease	*ctpA*	717	48.3	8.7
17	High affinity branched-chain amino acid ABC transporter, periplasmic amino acid-binding protein		420	39.9	8.94
	Hydrogenase expression/formation protein	*hypD*	151	40.9	8.59
18	Dihydrodipicolinate synthase	*dapA*	315	33	6.02
	Putative UDP-glucose 4-epimerase		214	35.6	7
	*O*-acetylserine sulfhydrylase B	*cysM*	201	32.5	6.62

## Discussion

Immunizing laying hens and subsequently collecting their eggs is a cheap and straightforward method to obtain high amounts of specific antibodies [6,11]. In our study, *C. jejuni*-specific IgY was dramatically induced in egg yolk of hens immunized with a *C. jejuni* whole-cell lysate, resulting in titers of up to 1:16 000 as determined by ELISA. In contrast, specific IgY in egg white and specific IgA and IgM in egg white and yolk were not significantly induced after immunization. This indicates that only transfer of IgY to egg yolk is biologically relevant in the overall transfer of immunoglobulins into eggs after immunization of hens, which is in line with Dohms et al. [20].

In the first in vivo trial, using the whole-cell lysate of *C. jejuni*, broiler chicks were provided with feed supplemented with (hyperimmune) egg yolks from day seven onward. At 10 days of age three out of 11 birds were orally inoculated with approximately 8 × 10^3^ cfu of *C. jejuni*. Colonization of these seeder animals is supposed to result in transmission of *C. jejuni* to the remainder of the birds. Three days after inoculation, cecal *C. jejuni* numbers of seeder birds receiving hyperimmune egg yolk were significantly reduced compared to control seeders without IgY (by over 5 logs). Moreover, none of the contact birds were colonized with *C. jejuni*, while chicks receiving control eggs carried high (ca. log_10_ 7 cfu/g) bacterial numbers in their ceca. It is not clear at what site (the cecum or more proximal in the GI tract) the IgY fraction was active, but clearly it captured ingested *C. jejuni* bacteria, disabling them to colonize and transmit to other birds. In addition, specific IgY increased bacterial binding of *C. jejuni* to chicken intestinal mucus, suggesting that it promotes bacterial uptake in the mucus layer for enhanced mucosal clearance. Because several of the identified immunodominant antigens are believed to be associated with the bacterial outer-membrane, a second (analogous) in vivo trial was performed using egg yolks containing IgY against only the hydrophobic protein fraction of *C. jejuni*. Although the effects observed in this second in vivo trial were less pronounced compared to the results obtained in the first in vivo trial using IgY against the whole-cell lysate of *C. jejuni* (probably due to the higher inoculation dose of the seeders (3 × 10^4^ cfu vs. 8 × 10^3^ cfu)), hyperimmune yolks reduced cecal bacterial counts of seeders by 2 log_10_ cfu and dramatically reduced *C. jejuni* transmission to contact birds, indicating that the hydrophobic protein fraction is important for immunization but that also non-hydrophobic proteins may be involved in this immune response. Previous passive immunization studies with *Salmonella enterica* by other researchers [21] indicated no significant effect on cecal colonization of broilers receiving feed supplemented with freeze-dried egg yolk powder containing anti-*Salmonella enterica* IgY. Cecal *Salmonella* counts in birds receiving feed supplemented with 5% (wt/wt) yolk powder on day 3 post-inoculation were, however, (non-significantly) reduced by three logs compared to control birds. These authors suggested that the antibodies were denatured and degraded along the GI tract, thereby reaching the ceca at insufficient concentrations, but it cannot be ruled out that a significant reduction could be achieved using more animals per group. In our study, egg yolks were administered as such to exclude degradation during freeze-drying. This might indicate that egg yolks form a protective matrix allowing IgY to survive the digestive enzymes and the low pH along the GI tract [11]. Other factors that may be responsible for the differential outcome between our study and that of Chalghoumi et al. [21] include (1) age of the animals at challenge, (2) experimental set-up, since we used a seeder experiment while all birds were challenged with *Salmonella*, (3) immunization protocol and (4) bacterium-specific differences between *Salmonella enterica* and *C. jejuni*.

We identified the most likely *C. jejuni* proteins responsible for the induction of specific IgY. Because a *C. jejuni* whole-cell lysate was used, specific IgY could be induced against both membrane-bound as well as cytoplasmic proteins. Although our obtained results are fairly consistent with those reported in other studies examining antigenic proteins of *C. jejuni*, there was a striking absence in our study of Omp18, Cme, Cja and PEB proteins, identified in another study [22]. These differences might be in part explained by *C. jejuni* strain differences. In addition, it is not clear whether repeated immunization of chickens in their breast muscle could lead to a different immune response compared to that observed during natural gut colonization with *C. jejuni*.

A first group of proteins that reacted with the purified egg yolk IgY are flagellar proteins: flagellar hook protein FlgE, major flagellin FlaA and minor flagellin FlaB. FlgE and FlaA are known to be immunogenic and are needed for full motility of *C. jejuni*[22,23]. In addition, FlgE mediates flagellar assembly and protein secretion in *C. jejuni*[22], while FlaA mutation may result in reduced colonization in chicks [24]. These results are consistent with Shoaf-Sweeney et al. [22] who reported the presence of maternal antibodies reacting with these flagellar proteins in the serum of laying hens. Despite these flagellar proteins, which were shown to be highly immunogenic in this study, specific IgY did not affect *C. jejuni* motility. The major outer-membrane protein (MOMP) of *C. jejuni*, encoded by the *porA* gene, was identified in several gel pieces, pointing to its abundance in the *C. jejuni* proteome [25]. In addition to being immunogenic [26], MOMP is involved in adhesion and transmembrane ion transport in *C. jejuni*[25]. However, MOMP is extremely genetically diverse and several conformational epitopes have been implicated in the induction of protective immunity [26], thereby possibly hindering its use in vaccine applications. Methyl-accepting chemotaxis (MCP) proteins are transmembrane receptors for chemotactic stimuli [23]. Attached to these transmembrane receptors is the *C. jejuni* chemotaxis protein V (CheV), a coupling protein involved in transducing chemotactic signals in *C. jejuni*[27], which was also identified in this study. Another immunodominant protein was the branched-chain amino acid ATP-binding cassette transport protein LivJ, a periplasmic binding protein probably involved in transporting amino acids into the bacterial cell [28] and crucial for chick colonization [22]. An additional role for LivJ in the interaction of *C. jejuni* with the chick cecum was suggested by Ribardo and Hendrixson [28]. ATP synthase is abundantly present and highly conserved among bacteria [29]. The protein is associated with the membrane where it performs its role in energy metabolism. In this study, the alpha subunit of ATP synthase F1 (AtpA) was found to be immunogenic in *C. jejuni*, as already reported for *C. concisus*[29].

Several of the identified antigens in this study were proteins mainly located in the bacterial cytoplasm where they perform their respective functions. Some of these antigens may nevertheless be interesting candidates for vaccine development. GroEL is an extremely conserved [30] 60-kDa heat shock protein that plays a crucial role in the *C. jejuni* stress response and has previously been shown to be an immunodominant antigen of *C. jejuni*[31]. Although mainly located in the bacterial cytoplasm, in *Salmonella* Typhymurium (and other bacteria) GroEL is suggested to be expressed on the cell surface as well since it has been shown to mediate *Salmonella* adhesion [32]. The highly conserved translation elongation factor thermo unstable (EF-Tu) was found to be abundantly present in the *C. jejuni* whole-cell lysate, which is in line with observations in other bacteria [33]. Despite its cytoplasmic role during protein synthesis, EF-Tu was shown to be translocated to the surface in several bacteria, where it mediates adhesion and invasion of host cells [33]. Immunization of mice with *Burkholderia pseudomallei* EF-Tu resulted in a potent immune response that was partially protective against melioidosis. Finally, a putative secreted carboxyl-terminal protease of *C. jejuni*, CtpA, was identified. This protein has been shown to be highly prevalent and conserved in *Burkholderia mallei*, another Gram-negative bacterium [34]. The exact function, as well as prevalence and conservation of CtpA in *C. jejuni* is less understood, but the immunodominance of this protein indicates CtpA might be an interesting candidate for subunit vaccin development.

This study reports some promising observations, but further research is needed before a commercial product can be promoted. First of all, it needs to be established whether this protective effect is maintained over periods exceeding three days post-inoculation. Second, the immunization protocol and the yolk dose in the feed need to be optimized. Further research will also have to elucidate whether this strategy is capable of providing cross-protection against heterologous *C. jejuni* strains. The increased mucosal adherence observed for at least one of the heterologous *C. jejuni* strains tested in this study, suggests that (partial) cross-protection may be attained, but in vivo trials are necessary to proof this hypothesis. Eventually, this may lead to the development of a passive immunization strategy that can successfully control *C. jejuni* in chickens. If the relevant protective antigens of *C. jejuni* could be identified and studies regarding conservation and prevalence among *C. jejuni* strains are conducted, subunit vaccines able to target a wide range of *C. jejuni* strains could be developed and used for immunization of hens in order to prepare yolks for passive immunization applications.

To conclude, we here demonstrate for the first time that feeding broilers IgY-rich yolks from hens immunized with *C. jejuni* dramatically reduces both *C. jejuni* numbers in the ceca after challenge with the homologous strain and transmission to non-inoculated contact birds, providing a solid base for further research regarding passive immunization to control *C. jejuni* colonization in broiler flocks.

## Competing interests

The authors declare that they have no competing interests.

## Authors’ contributions

DH participated in the design of the study, performed in vivo and in vitro experiments, analysed data and drafted the manuscript. KVS performed LC-MS/MS analyses and coordinated the immunoproteomics experiments. EV assisted in the in vitro experiments. MV and AM participated in the coordination and analyses of the in vivo trials. TS and LDZ provided the heterologous *C. jejuni* strains. TS assisted in the MLST analysis. MH edited the manuscript. LDZ, FH and DD participated in the coordination of the study. FH and DD edited the manuscript. FP coordinated and participated in the design of the study, helped to interpret the results and edited the manuscript. All authors read and approved the final manuscript.

## References

[B1] EFSA The European Union summary report on trends and sources of zoonoses, zoonotic agents and food-borne outbreaks in 2009 EFSA J 2011 9 2090 10.2903/j.efsa.2018.5500 PMC7009540 32625785

[B2] Hermans D Van Deun K Messens W Martel A Van Immerseel F Haesebrouck F Rasschaert G Heyndrickx M Pasmans F *Campylobacter* control in poultry by current intervention measures ineffective: urgent need for intensified fundamental research Vet Microbiol 2011 152 219 228 10.1016/j.vetmic.2011.03.010 21482043

[B3] van Gerwe T Miflin JK Templeton JM Bouma A Wagenaar JA Jacobs-Reitsma WF Stegeman A Klinkenberg D Quantifying transmission of *Campylobacter jejuni* in commercial broiler flocks Appl Environ Microbiol 2009 75 625 628 10.1128/AEM.01912-08 19047389 PMC2632138

[B4] Chalghoumi R Hen egg yolk antibodies (IgY), production and use for passive immunization against bacterial enteric infections in chicken: a review Biotechnol Agron Soc Environ 2009 13 295 308

[B5] Cawthraw SA Newell DG Investigation of the presence and protective effects of maternal antibodies against *Campylobacter jejuni* in chickens Avian Dis 2010 54 86 93 10.1637/9004-072709-Reg.1 20408404

[B6] Dias da Silva W Tambourgi DV IgY: a promising antibody for use in immunodiagnostics and immunotherapy Vet Immunol Immunopathol 2010 135 173 180 10.1016/j.vetimm.2009.12.011 20083313 PMC7126787

[B7] Tsubokura K Berndtson E Bogstedt A Kaijser B Kim M Ozeki M Hammarstrom L Oral administration of antibodies as prophylaxis and therapy in *Campylobacter jejuni*-infected chickens Clin Expl Immunol 1997 108 451 455 10.1046/j.1365-2249.1997.3901288.x PMC1904686 9182891

[B8] Woldemariam E Bouma A Vernooij JC Stegeman A The sensitivity and specificity of fecal and cecal culture for the detection of *Campylobacter* in Dutch broiler flocks quantified by Bayesian analysis Int J Food Microbiol 2008 121 308 312 10.1016/j.ijfoodmicro.2007.11.011 18068250

[B9] Smith DJ King WF Godiska R Passive transfer of immunoglobulin Y antibody to *Streptococcus* mutans glucan binding protein B can confer protection against experimental dental caries Infect Immun 2001 69 3135 3142 10.1128/IAI.69.5.3135-3142.2001 11292733 PMC98269

[B10] Shimamoto C Tokioka S Hirata I Tani H Ohishi H Katsu K Inhibition of *Helicobacter pylori* infection by orally administered yolk-derived anti-*Helicobacter pylori* antibody Hepatogastroenterology 2002 49 709 714 12063975

[B11] Mine Y Kovacs-Nolan J Chicken egg yolk antibodies as therapeutics in enteric infectious disease: a review J Med Food 2002 5 159 169 10.1089/10966200260398198 12495588

[B12] Hermans D Martel A Van Deun K Van Immerseel F Heyndrickx M Haesebrouck F Pasmans F The cinnamon-oil ingredient *trans*-cinnamaldehyde fails to target *Campylobacter jejuni* strain KC40 in the broiler chicken cecum despite marked *in vitro* activity J Food Prot 2011 74 1729 1734 10.4315/0362-028X.JFP-10-487 22004822

[B13] Van Deun K Pasmans F Ducatelle R Flahou B Vissenberg K Martel A Van den Broeck W Van Immerseel F Haesebrouck F Colonization strategy of *Campylobacter jejuni* results in persistent infection of the chicken gut Vet Microbiol 2008 130 285 297 10.1016/j.vetmic.2007.11.027 18187272

[B14] Bird CR Thorpe R Walker JM Purification of immunoglobulin Y (IgY) from chicken eggs The Protein Protocols Handbook. Part VII 2002 Totowa, NJ: Humana Press Inc. 1009–1011

[B15] Dingle KE Colles FM Wareing DRA Ure R Fox AJ Bolton FE Bootsma HJ Willems RJL Urwin R Maiden MCJ Multilocus sequence typing system for *Campylobacter jejuni* J Clin Microbiol 2001 39 14 23 10.1128/JCM.39.1.14-23.2001 11136741 PMC87672

[B16] *Campylobacter* MLST homepage [http://pubmlst.org/campylobacter]

[B17] Van Steendam K Tilleman K De Ceuleneer M De Keyser F Elewaut D Deforce D Citrullinated vimentin as an important antigen in immune complexes from synovial fluid of rheumatoid arthritis patients with antibodies against citrullinated proteins Arthritis Res Ther 2010 12 R132 10.1186/ar3070 20609218 PMC2945022

[B18] Cheung KJ Tilleman K Deforce D Colle I Van Vlierberghe H The HCV serum proteome: a search for fibrosis protein markers J Viral Hepat 2009 16 418 429 10.1111/j.1365-2893.2009.01083.x 19226329

[B19] Van Steendam K De Ceuleneer M Dhaenens M Van Hoofstat D Deforce D Mass spectrometry-based proteomics as a tool to identify biological matrices in forensic science Int J Legal Med 2013 127 287 298 10.1007/s00414-012-0747-x 22843116 PMC3578717

[B20] Dohms JE Saif YM Bacon WL Metabolism and passive transfer of immunoglobulins in the turkey hen Am J Vet Res 1978 39 1472 1481 697159

[B21] Chalghoumi R Marcq C Théwis A Portetelle D Beckers Y Effects of feed supplementation with specific hen egg yolk antibody (immunoglobulin Y) on *Salmonella* species cecal colonization and growth performances of challenged broiler chickens Poult Sci 2009 88 2081 2092 10.3382/ps.2009-00173 19762860

[B22] Shoaf-Sweeney KD Larson CL Tang X Konkel ME Identification of *Campylobacter jejuni* proteins recognized by maternal antibodies of chickens Appl Environ Microbiol 2008 74 6867 6875 10.1128/AEM.01097-08 18805999 PMC2583476

[B23] Hermans D Pasmans F Heyndrickx M Van Immerseel F Martel A Van Deun K Haesebrouck F A tolerogenic mucosal immune response leads to persistent *Campylobacter jejuni* colonization in the chicken gut Crit Rev Microbiol 2012 38 17 29 10.3109/1040841X.2011.615298 21995731

[B24] Wassenaar TM Van der Zeijst BA Ayling R Newell DG Colonization of chicks by motility mutants of *Campylobacter jejuni* demonstrates the importance of flagellin A expression J Gen Microbiol 1993 139 1171 1175 10.1099/00221287-139-6-1171 8360610

[B25] Islam A Raghupathy R Albert MJ Recombinant PorA, the major outer membrane protein of *Campylobacter jejuni*, provides heterologous protection in an adult mouse intestinal colonization model Clin Vaccine Immunol 2010 17 1666 1671 10.1128/CVI.00255-10 20861330 PMC2976099

[B26] Huang S Sahin O Zhang Q Infection-induced antibodies against the major outer membrane protein of *Campylobacter jejuni* mainly recognize conformational epitopes FEMS Microbiol Lett 2007 272 137 143 10.1111/j.1574-6968.2007.00752.x 17521366

[B27] Lertsethtakarn P Ottemann KM Hendrixson DR Motility and chemotaxis in *Campylobacter* and *Helicobacter* Annu Rev Microbiol 2011 65 389 410 10.1146/annurev-micro-090110-102908 21939377 PMC6238628

[B28] Ribardo DA Hendrixson DR Analysis of the LIV system of *Campylobacter jejuni* reveals alternative roles for LivJ and LivK in commensalism beyond branched-chain amino acid transport J Bacteriol 2011 193 6233 6243 10.1128/JB.05473-11 21949065 PMC3209225

[B29] Kovach Z Kaakoush NO Lamb S Zhang L Raftery MJ Mitchell H Immunoreactive proteins of *Campylobacter concisus*, an emergent intestinal pathogen FEMS Immunol Med Microbiol 2011 63 387 396 10.1111/j.1574-695X.2011.00864.x 22092566

[B30] Thies FL Weishaupt A Karch H Hartung HP Giegerich G Cloning, sequencing and molecular analysis of the *Campylobacter jejuni* groESL bicistronic operon Microbiology 1999 145 89 98 10.1099/13500872-145-1-89 10206714

[B31] Zhang M Meng F Cao F Qiao B Liu G Liu H Zhou Y Dong H Gu Y Xiao D Zhang Y Zhang J Cloning, expression, and antigenicity of 14 proteins from *Campylobacter jejuni* Foodborne Pathog Dis 2012 9 706 712 10.1089/fpd.2011.1122 22779748

[B32] Tsugawa H Ito H Ohshima M Okawa Y Cell adherence-promoted activity of *Plesiomonas shigelloides* groEL J Med Microbiol 2007 56 23 29 10.1099/jmm.0.46766-0 17172512

[B33] Nieves W Heang J Asakrah S HönerzuBentrup K Roy CJ Morici LA Immunospecific responses to bacterial elongation factor Tu during *Burkholderia* infection and immunization PloS One 2010 5 e14361 10.1371/journal.pone.0014361 21179405 PMC3003680

[B34] Bandara AB DeShazre D Inzana TJ Sriranganathan N Schurig GG Boyle SM A disruption of *ctpA* encoding carboxy-terminal protease attenuates *Burkholderia mallei* and induces partial protection in CD1 mice Microb Pathog 2008 45 207 216 10.1016/j.micpath.2008.05.005 18614331

